# Intravitreal Bevacizumab and Triamcinolone for Treatment of Cystoid Macular Oedema Associated with Chronic Myeloid Leukaemia and Imatinib Therapy

**DOI:** 10.1155/2015/713868

**Published:** 2015-02-04

**Authors:** Eric K. Newcott, Abdallah A. Ellabban, Shokufeh Tavassoli, Ahmed Sallam

**Affiliations:** Department of Ophthalmology, Gloucester Royal Hospital, Gloucester GL1 3NN, UK

## Abstract

*Purpose.* To evaluate the efficacy of intravitreal bevacizumab and triamcinolone in the treatment of cystoid macular oedema in a case with chronic myeloid leukaemia on imatinib treatment. *Methods.* We treated a 78-year-old man with bilateral cystoid macular oedema with intravitreal triamcinolone and subsequent bevacizumab in one eye and intravitreal bevacizumab, alone, in the fellow eye. *Results.* Serial intravitreal bevacizumab with and without triamcinolone treated cystoid macular oedema in both eyes and improved the vision. *Conclusion.* Intravitreal bevacizumab and triamcinolone could be viable options to treat cystoid macular oedema due to chronic myeloid leukaemia and imatinib therapy.

## 1. Introduction

A 78-year-old man was referred to our department by his optometrist because of bilateral gradual decline in his vision that has progressed over 10 months. Past medical history was consistent with a diagnosis of chronic myeloid leukemia (CML) about 9 months before his ocular presentation. This was treated with imatinib mesylate, a selective inhibitor of BCR-ABL tyrosine kinase, commenced 1 month after the diagnosis of CML. His CML was favourably controlled on imatinib with no obvious drug-related side effects apart from mild periorbital oedema and two isolated episodes of scalp oedema that started after commencing imatinib and resolved without treatment.

**Figure 1:**
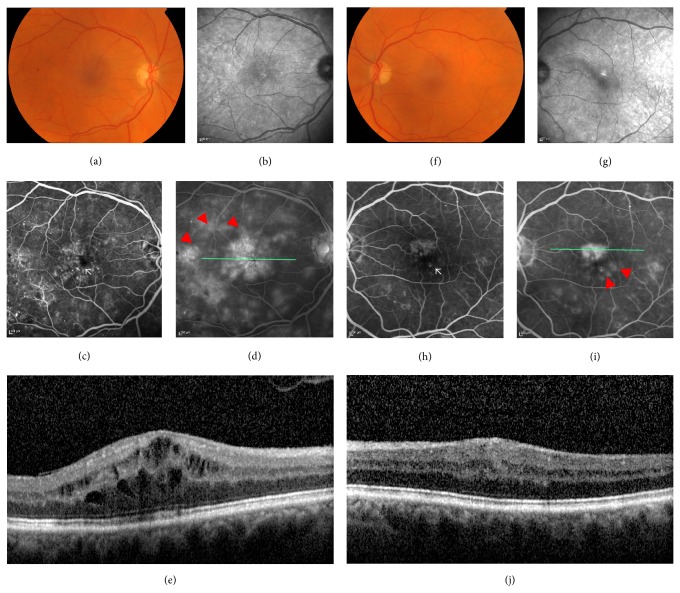
A composite of colour, infrared photographs, early and late fluorescein angiogram frames, and spectral domain optical coherence tomography (OCT) scan of the right eye (left column (a)–(e)) and left eye (right column (f)–(j)). Note the irregularities of the foveal avascular zone (white arrow) and the areas of vascular leakage at the macula (red arrowheads). The green line indicates the position of the OCT scan.

At his first ophthalmic review, his best corrected visual acuity was 0.6 Logarithm of the minimum angle of resolution (LogMAR) in the right eye and 0.2 in the left. He had bilateral cystoid macular oedema (CMO) with the right being worse than the left with some scattered microaneurysms and perifoveal telangiectasia. Fluorescein angiography confirmed the presence of macular oedema and showed some irregularities of both foveal avascular zones suggesting associated macular ischaemia (Figure 1).

**Figure 2:**
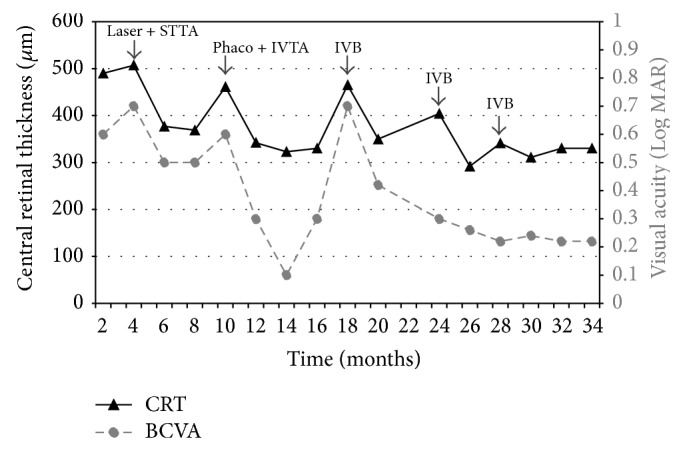
Visual acuity and central retinal thickness changes with treatment over time. BCVA = best corrected visual acuity; CRT = central retinal thickness; LogMAR = Logarithm of the minimum angle of resolution; Phaco = phacoemulsification; IVB = intravitreal bevacizumab; IVTA = intravitreal triamcinolone acetonide; STTA = sub-Tenon triamcinolone acetonide.

A subsequent optical coherence tomography (OCT) scan showed worsening of right macular oedema, whilst the left eye had settled down spontaneously. Macular oedema and vision improved to 0.3 LogMAR following sequential treatments with a sub-Tenon's injection of triamcinolone acetonide (TA) and focal macular laser to the microaneurysms in the right eye (Figure 2).

After 4 months, bilateral CMO recurred and was persistently worse in the right eye compared to the left eye. There was also a concurrent right mild posterior subcapsular cataract that had developed over the course of his followup, contributing to his diminished visual acuity of 0.5 LogMAR. To treat both conditions, the cataract and the CMO, he underwent phacoemulsification with an intraocular lens implant and an intravitreal injection of 4 mg of TA. This resulted in complete resolution of macular oedema and improvement of visual acuity to 0.1 LogMAR. At this time, the left macula began to develop oedema with only minimal cataract present. The patient was offered a treatment with a standard dose of 1.25 mg/0.05 mL of intravitreal bevacizumab (Avastin). This improved the visual acuity to 0.1 LogMAR in the left eye, and the macular oedema settled. Subsequent to the bevacizumab treatment in the left eye, his right eye redeveloped CMO and was also treated with an injection of bevacizumab. Thirty-four months since his ocular presentation, he has had a total of one triamcinolone acetonide injection and 3 bevacizumab injections to the right eye in addition to 1 bevacizumab treatment to the left eye. Best corrected visual acuity remained stable at 0.2 LogMAR in the right eye and 0.1 LogMAR in the left eye with a repeat pattern of complete CMO resolution after each injection (Figure 2).

## 2. Discussion

In the current literature, the management of cystoid macular oedema in CML is limited to only a few case reports [1–4]. It should be noted that the majority of these reports are of patients with CML concurrently on imatinib mesylate therapy, which rarely has been associated with CMO [2, 3].

In 1996, Horgan et al. reported a patient who presented with CML related CMO and a decreased vision to about 1.0 LogMAR [1]. In their patient, oral acetazolamide therapy was used to treat the macular oedema and full resolution occurred when the patient underwent a bone marrow transplant. There are a few case reports detailing the presence of macular oedema in patients concurrently taking imatinib mesylate. These cases differ from our patient in that the macular oedema was not accompanied with ischaemic macular changes [2–4]. In one patient, the oedema consisted of an isolated collection of subretinal fluid [2], while two other patients developed cystoid macular oedema [3, 4]. Out of the two cases of cystoid macular oedema, one case was reported early in the postoperative period following phacoemulsification surgery whilst being on imatinib [3]. In the other case report, short-term improvement of the cystoid macular oedema was demonstrated after a tapered course of oral prednisolone, but resolution occurred only when the imatinib was discontinued [4].

Only one previous case report exists, detailing ischaemic maculopathy in a patient with CML whilst being on imatinib therapy [5]. In this case, the patient noted a central scotoma and had retinal signs of multiple cotton wool spots, haemorrhages, telangiectasia, and macular ischaemia. No macular oedema was noted, however, and the scotoma did not improve when the imatinib mesylate was discontinued.

Although the retinal appearance in our case is suggestive of leukaemic pathology given the areas of capillary leakage and foveal avascular zone disruption that was noted on fluorescein angiogram in addition to the CMO, it is known that imatinib can cause fluid retention and so the bilateral CMO could also be the consequences of imatinib therapy especially when the development of CMO is very rare in CML.

In summary, there is a paucity of literature on the treatment of CMO in patients with CML. In this case report, we demonstrated that resolution of macular oedema and visual improvement can be achieved with intravitreal steroids and antivascular endothelial growth factor treatment, albeit the need for repeated treatments based on clinical and OCT findings.
